# Supplementary material for: A meta learning and task adaptive approach for drug target affinity prediction
Source: Nat Commun. 2026 Mar 10;17:3734. doi: 10.1038/s41467-026-70554-5 (PMC13102954; doi:10.1038/s41467-026-70554-5)
Supplement: Supplementary file 1 — Supplementary Information [file 41467_2026_70554_MOESM1_ESM.docx]

## Supplementary Information

## A meta learning and task adaptive approach for drug target affinity prediction

Mengxuan Wan^1,2,†^, Yanpeng Zhao^1,†^, Yixin Zhang^2,†^, Huiyan Xu^2,3^, Duoyun Yi^2,3^, Peng Zan^1,3,∗^, Song He^2,∗^, Xiaochen Bo^2,∗^

^1^ School of Medicine, Shanghai University, Shanghai 200444, China

^2^ Academy of Military Medical Sciences, Beijing 100850, China

^3^ Shanghai Key Laboratory of Power Station Automation Technology, School of Mechatronics Engineering and Automation, Shanghai University, Shanghai 200444, China

* Corresponding authors.

† These authors contributed equally to this work.

E-mail address: boxc@bmi.ac.cn (Xiaochen Bo), hes1224@163.com (Song He), zanpeng@shu.edu.cn (Peng Zan)

**Supplementary Table 1. The statistical information of BindingDB, KIBA, and Davis datasets**

| **DataSet** | **Number of proteins** | **Number of drugs** | **Dataset size** |
| --- | --- | --- | --- |
| BindingDB | 1088 | 9862 | 42203 |
| Kiba | 229 | 2068 | 118254 |
| Davis | 379 | 68 | 30056 |

**Supplementary Table 2. The statistical information of the LIT-PCBA dataset**

| **Target** | **Drugs** | **Active** | **Inactives** | **Active rate(%）** |
| --- | --- | --- | --- | --- |
| Beta2 adrenergic receptor | Agonists | 17 | 311748 | 0.054 |
| Aldehyde dihydrogenase 1 | Inhibitors | 5363 | 101874 | 5.264 |
| Estrogen receptor α | Agonists | 13 | 4378 | 0.296 |
| Estrogen receptor α | Antagonists | 88 | 3820 | 2.303 |
| FLAP Endonuclease | Inhibitors | 360 | 350718 | 0.102 |
| Glucocerebrosidrase | Inhibitors | 163 | 291241 | 0.055 |
| Isocitrate dihydrogenase | Inhibitors | 39 | 358757 | 0.010 |
| Histone acetyltransferase KAT2A | Inhibitors | 194 | 342729 | 0.056 |
| Mitogen-activated protein kinase 1 | Inhibitors | 308 | 61567 | 0.500 |
| Mechanistic target of rapamycin | Inhibitors | 97 | 32972 | 0.294 |
| Kappa opioid receptor | Agonists | 24 | 269475 | 0.008 |
| Pyruvate kinase muscle isoform 2 | Inhibitors | 546 | 244679 | 0.223 |
| Peroxisome proliferator-activated receptor γ | Inhibitors | 24 | 4071 | 0.593 |
| Cellular tumor antigen p53 | Inhibitors | 64 | 3345 | 1.913 |
| Vitamin D receptor | Inhibitors | 655 | 262648 | 0.249 |

**Supplementary Table 3. Comparison of Cross-domain Transfer Performance on KIBA→BindingDB**

| **Methods** | **MSE** | **CI** | **R2** | **Spearman** | **Pearson** |
| --- | --- | --- | --- | --- | --- |
| ColdDTA | 0.9937  (0.0380) | 0.7213  (0.0217) | 0.2842  (0.1054) | 0.4630  (0.0835) | 0.5503  (0.0679) |
| Co-VAE | 1.0283  (0.2440) | 0.6853  (0.0486) | 0.2176  (0.0787) | 0.3834  (0.1395) | 0.5212  (0.0914) |
| HiSIF | 1.2328  (0.1207) | 0.7209  (0.0305) | 0.2772  (0.1366) | 0.4387  (0.1081) | 0.5280  (0.0982) |
| PSICHIC | 0.9604  (0.1513) | 0.7359  (0.0452) | 0.3066  (0.1180) | 0.5182  (0.0587) | 0.5695  (0.0957) |
| DeepDTA | 2.5974  (0.2652) | 0.6024  (0.0401) | -0.2424  (0.2709) | 0.2623  (0.1381) | 0.1882  (0.1331) |
| MetaDTA | 1.1047  (0.4644) | **0.7592**  **(0.0436)** | 0.2824  (0.2743) | 0.4936  (0.0935) | 0.5639  (0.1254) |
| CML | 1.9214  (0.0694) | 0.7000  (0.0248) | 0.1847  (0.1036) | 0.3526  (0.1149) | 0.3845  (0.1480) |
| ZeroBind | **0.9598**  **(0.2069)** | 0.7241  (0.0423) | 0.2239  (0.0705) | 0.4613  (0.1251) | 0.5230  (0.0994) |
| AdaMBind | 0.9623  (0.1003) | 0.7458  (0.0374) | **0.3446**  **(0.1410)** | **0.5326**  **(0.1244)** | **0.5961**  **(0.1019)** |

The best results are highlighted in bold, while the second-best results are underlined. Five independent replications of each method were performed (n = 5). Data are expressed as means (std).

**Supplementary Table 4. Performance of AdaMBind on LIT-PCBA**

|  | EF@1% | EF@5% | Precision@10% | Precision@20% | BEDROC(80.5) |
| --- | --- | --- | --- | --- | --- |
| TP53 | 4.1966 | 3.6875 | 0.1000 | 0.1000 | 9.3461 |
| ESR | 4.2080 | 4.3959 | 0.1000 | 0.1500 | 11.4917 |
| ALDH1 | 1.0492 | 1.0544 | 0.1000 | 0.0500 | 5.2929 |
| KAT2A | 2.7939 | 1.2290 | 0.0000 | 0.0000 | 2.6784 |
| MTORC1 | 2.4430 | 0.7320 | 0.0000 | 0.0000 | 1.7143 |
| MAPK1 | 1.7070 | 1.5700 | 0.0000 | 0.0000 | 2.3031 |

**Supplementary Table 5. Performance on the LIT-PCBA Dataset as Reported by Zhou and Lan**

| **Methods** | **BEDROC (%)** | **EF @1%** | **EF @5%** |
| --- | --- | --- | --- |
| ROCS | – | 2.48 | – |
| Phase Shape | – | 2.98 | – |
| LIGSIFT | – | 2.39 | – |
| SHAFTS | – | 2.79 | – |
| Surflex | – | 2.50 | – |
| Glide-SP | 4.00 | 3.41 | 2.01 |
| Planet | – | 3.87 | 2.43 |
| Gnina | 5.40 | 4.63 | – |
| DeepDTA | 2.53 | 1.47 | – |
| BigBind | – | 3.82 | – |
| DrugCLIP | 6.23 | 5.51 | 2.27 |
| S-MolSearch₀.₄ | 7.58 | 6.28 | 2.47 |
| S-MolSearch₀.₉ | 8.48 | 7.36 | 3.21 |
| SaBAN | 13.35 | 6.33 | 3.36 |

**Supplementary Table 6. EF@1% on the LIT-PCBA Dataset as Reported by Morris**

| **Targets** | **Vina** | **rDock** | **Plants** | **LeDock** | **AutoDock4** | **Naïve Consensus** | **XGBoost** | **MLP** | **Ensemble MLP** |
| --- | --- | --- | --- | --- | --- | --- | --- | --- | --- |
| ADRB2 | 0.00 | 0.00 | 0.00 | 0.00 | 0.00 | 5.88 | 0.00 | 11.77 | 5.88 |
| ALDH1 | 1.73 | 1.70 | 1.66 | 1.62 | 2.11 | 1.97 | 0.56 | 0.95 | 0.97 |
| GBA | 4.91 | 3.07 | 4.91 | 1.84 | 5.52 | 1.84 | 4.30 | 4.91 | 7.98 |
| IDH1 | 2.56 | 2.56 | 2.56 | 5.13 | 0.00 | 0.00 | 2.56 | 5.13 | 2.56 |
| MEAN | 2.30 | 1.83 | 2.28 | 2.15 | 1.91 | 2.42 | 1.85 | 5.69 | 4.35 |
| STD | 2.04 | 1.35 | 2.05 | 2.15 | 2.61 | 2.48 | 1.96 | 4.48 | 3.17 |

**Supplementary Table 7. BEDROC(α=80.5) on the LIT-PCBA Dataset as Reported by Morris**

| **Targets** | **Vina** | **rDock** | **Plants** | **LeDock** | **AutoDock** | **Naïve Consensus** | **XGBoost** | **MLP** | **Ensemble MLP** |
| --- | --- | --- | --- | --- | --- | --- | --- | --- | --- |
| ADRB2 | 0.000 | 0.000 | 0.000 | 0.000 | 0.000 | 0.027 | 0.000 | 0.091 | 0.057 |
| ALDH1 | 0.094 | 0.088 | 0.087 | 0.084 | 0.113 | 0.100 | 0.038 | 0.047 | 0.049 |
| GBA | 0.029 | 0.018 | 0.032 | 0.006 | 0.038 | 0.016 | 0.035 | 0.042 | 0.047 |
| IDH1 | 0.025 | 0.009 | 0.007 | 0.026 | 0.000 | 0.000 | 0.015 | 0.029 | 0.008 |
| MEAN | 0.037 | 0.029 | 0.031 | 0.029 | 0.038 | 0.036 | 0.022 | 0.052 | 0.040 |
| STD | 0.040 | 0.040 | 0.039 | 0.038 | 0.053 | 0.044 | 0.018 | 0.027 | 0.022 |

**Supplementary Table 8. Model performance with different drug encoders**

| **Dataset** | **GNN** | **Results** | | |
| --- | --- | --- | --- | --- |
|  |  | **MSE** | **CI** | **R2** |
| BindingDB | GCN | 0.8601 | 0.7820 | 0.4614 |
|  | GAT | 0.9125 | 0.7733 | 0.4063 |
|  | GIN | 0.8818 | 0.7867 | 0.4213 |
|  | GAT+GCN | 0.8578 | 0.7843 | 0.4815 |
| Kiba | GCN | 0.4295 | 0.7545 | 0.4285 |
|  | GAT | 0.4792 | 0.7226 | 0.3432 |
|  | GIN | 0.4782 | 0.7328 | 0.3473 |
|  | GAT+GCN | 0.4452 | 0.7432 | 0.3922 |
| Davis | GCN | 0.6252 | 0.8037 | 0.4227 |
|  | GAT | 0.6196 | 0.8016 | 0.4169 |
|  | GIN | 0.5961 | 0.8032 | 0.4326 |
|  | GAT+GCN | 0.5060 | 0.8222 | 0.4693 |

For the key hyperparameters of each major component (e.g., noise, outer learning rate, inner learning rate, Batch size in support set, number of candidate tasks$B$, Number of selected tasks$N_{slct}$, etc.), we adopted a grid search approach to explore different hyperparameter combinations. First, we conducted a series of experiments to jointly evaluate the influence of the inner learning rate and the batch size in the support set on model performance. Second, we explored the combined effect of the outer learning rate and the number of candidate tasks$B$. Then, we examined how the number of selected tasks $N_{slct}$ and noise impacts the model performance (MSE).

**Supplementary Table 9. Model performance with different drug encoders**

| **Hyper parameters** | **range** |
| --- | --- |
| noise | 0.1, 0.2, 0.3, 0.4, **0.5**, 0.6 |
| Inner learning rate | 1e-3, 5e-4, **1e-4**, 5e-5, 1e-5 |
| Outer learning rate | 5e-4, 1e-4, 5e-5, **1e-5** |
| Number of candidate tasks$B$ | 12, 13, 14, **15**, 16 |
| Number of selected tasks$N_{slct}$ | 4, 6, **8**, 10 |
| Batch size in the support set | 2, 4, **8,** 16 |

**Supplementary Table 10. Performance on Davis in random task split (Majority)**

| **Methods** | **MSE** | **CI** | **R2** | **Spearman** | **Pearson** |
| --- | --- | --- | --- | --- | --- |
| DeepDTA | 0.5487  (0.2057) | 0.7979  (0.0090) | 0.4096  (0.0575) | 0.5814  (0.0522) | 0.6567  (0.0422) |
| ColdDTA | 0.5248  (0.1913) | 0.8099  (0.0169) | 0.4348  (0.0533) | 0.6093  (0.0619) | 0.6637  (0.0427) |
| Co-VAE | 0.4217  (0.0961) | 0.7857  (0.0239) | 0.4137  (0.0815) | 0.5329  (0.0492) | 0.6524  (0.0466) |
| HiSIF | 0.4097  (0.1212) | 0.8194  (0.0209) | **0.5096**  **(0.0400)** | 0.6118  (0.0492) | **0.6946**  **(0.0466)** |
| MetaDTA | **0.3494**  **(0.1451)** | 0.8084  (0.0268) | 0.3811  (0.0393) | 0.5376  (0.0422) | 0.6205  (0.0420) |
| CML | 0.6926  (0.2690) | 0.7567  (0.0138) | 0.2593  (0.0344) | 0.5063  (0.0440) | 0.5221  (0.0393) |
| PSICHIC | 0.4299  (0.1549) | 0.8142  (0.0234) | 0.4646  (0.0808) | 0.6037  (0.0527) | 0.6624  (0.0456) |
| ZeroBind | 0.5923  (0.1021) | 0.7637  (0.0336) | 0.3448  (0.0374) | 0.5179  (0.0446) | 0.5489  (0.0362) |
| AdaMBind(ours) | 0.5060  (0.1869) | **0.8222**  **(0.0178)** | 0.4693  (0.0602) | **0.6257**  **(0.0435)** | 0.6878  (0.0430) |

The best results are highlighted in bold, while the second-best results are underlined. Five independent replications of each method were performed (n = 5). Data are expressed as means (std).

**Supplementary Table 11. Performance on KIBA in random task split (Majority)**

| **Methods** | **MSE** | **CI** | **R2** | **Spearman** | **Pearson** |
| --- | --- | --- | --- | --- | --- |
| DeepDTA | 0.5427  (0.0528) | 0.6806  (0.0105) | 0.2776  (0.0296) | 0.4779  (0.0258) | 0.5386  (0.0222) |
| ColdDTA | 0.4868  (0.0375) | 0.7115  (0.0106) | 0.3512  (0.0184) | 0.5541  (0.0236) | 0.5960  (0.0118) |
| Co-VAE | 0.5706  (0.0744) | 0.6753  (0.0213) | 0.2342  (0.0325) | 0.4610  (0.0528) | 0.5242  (0.0227) |
| HiSIF | 0.5268  (0.0553) | 0.6785  (0.0222) | 0.2849  (0.0506) | 0.4749  (0.0634) | 0.5434  (0.0437) |
| MetaDTA | 0.5648  (0.0852) | 0.6747  (0.0132) | 0.2308  (0.0213) | 0.3368  (0.0335) | 0.3637  (0.0361) |
| CML | 0.6734  (0.0901) | 0.6549  (0.0370) | 0.0879  (0.0733) | 0.4196  (0.0910) | 0.3597  (0.0615) |
| PSICHIC | 0.5592  (0.0667) | 0.7077  (0.0139) | 0.2773  (0.0227) | 0.5409  (0.0365) | 0.5404  (0.0237) |
| ZeroBind | 0.5727  (0.0395) | 0.6755  (0.0270) | 0.2484  (0.0239) | 0.4562  (0.0351) | 0.4810  (0.0331) |
| AdaMBind(ours) | **0.4295**  **(0.0566)** | **0.7545**  **(0.0245)** | **0.4285**  **(0.0392)** | **0.6528**  **(0.0545)** | **0.6559**  **(0.0297)** |

The best results are highlighted in bold, while the second-best results are underlined. Five independent replications of each method were performed (n = 5). Data are expressed as means (std).

**Supplementary Table 12. Performance on BindingDB in random task split (Majority)**

| **Methods** | **MSE** | **CI** | **R2** | **Spearman** | **Pearson** |
| --- | --- | --- | --- | --- | --- |
| DeepDTA | 1.1268  (0.3414) | 0.7513  (0.0477) | 0.3443  (0.1504) | 0.5486  (0.0948) | 0.5594  (0.1091) |
| ColdDTA | 1.0833  (0.1573) | 0.7540  (0.0165) | 0.3513  (0.0494) | 0.5919  (0.0246) | 0.5956  (0.0414) |
| Co-VAE | 1.2379  (0.4839) | 0.7412  (0.0789) | 0.3231  (0.1688) | 0.4257  (0.1433) | 0.4713  (0.1405) |
| HiSIF | 1.1076  (0.2574) | 0.7363  (0.0337) | 0.3317  (0.0644) | 0.5817  (0.0464) | 0.6387  (0.0503) |
| MetaDTA | 0.8636  (0.1597) | 0.7776  (0.0461) | 0.4186  (0.0506) | 0.5991  (0.0371) | 0.6530  (0.0374) |
| CML | 1.0017  (0.2407) | 0.7595  (0.0457) | 0.3946  (0.1183) | 0.5735  (0.0973) | 0.6392  (0.0940) |
| PSICHIC | 1.0048  (0.1011) | 0.7758  (0.0304) | 0.3119  (0.0636) | 0.5732  (0.0707) | 0.5821  (0.0653) |
| ZeroBind | 1.2613  (0.1437) | 0.7379  (0.0323) | 0.3491  (0.0511) | 0.5675  (0.0458) | 0.6176  (0.0517) |
| AdaMBind(ours) | **0.8578**  **(0.1077)** | **0.7843**  **(0.0221)** | **0.4815**  **(0.0657)** | **0.6578**  **(0.0413)** | **0.6966**  **(0.0430)** |

The best results are highlighted in bold, while the second-best results are underlined. Five independent replications of each method were performed (n = 5). Data are expressed as means (std).

**Supplementary Table 13. Performance on Davis in random task split (Few-shot)**

| **Methods** | **MSE** | **CI** | **R2** | **Spearman** | **Pearson** |
| --- | --- | --- | --- | --- | --- |
| DeepDTA | 0.6363  (0.0249) | **0.7693**  **(0.0098)** | 0.2062  (0.0079) | **0.5032**  **(0.0232)** | 0.5279  (0.0214) |
| ColdDTA | 0.7073  (0.0725) | 0.6997  (0.0126) | 0.1210  (0.0995) | 0.3772  (0.0269) | 0.4548  (0.0640) |
| Co-VAE | 0.6856  (0.0377) | 0.6896  (0.0301) | 0.1013  (0.0659) | 0.3561  (0.0572) | 0.4075  (0.0573) |
| HiSIF | 0.5877  (0.0348) | 0.7613  (0.0109) | 0.2495  (0.0312) | 0.4908  (0.0259) | 0.5232  (0.0317) |
| MetaDTA | 0.5363  (0.0753) | 0.7618  (0.0309) | 0.2213  (0.0499) | 0.4794  (0.0467) | 0.5099  (0.0375) |
| CML | 0.8138  (0.1723) | 0.6474  (0.0204) | 0.0689  (0.0589) | 0.2874  (0.0510) | 0.2728  (0.0708) |
| PSICHIC | 0.6539  (0.0586) | 0.7232  (0.0372) | 0.1634  (0.0449) | 0.4166  (0.0444) | 0.4525  (0.0327) |
| ZeroBind | 0.7971  (0.0615) | 0.6603  (0.0351) | 0.1059  (0.0465) | 0.3135  (0.0422) | 0.3335  (0.0412) |
| AdaMBind(ours) | **0.4744**  **(0.0216)** | 0.7507  (0.0450) | **0.2732**  **(0.0314)** | 0.4904  (0.0205) | **0.5499**  **(0.0187)** |

The best results are highlighted in bold, while the second-best results are underlined. Five independent replications of each method were performed (n = 5). Data are expressed as means (std).

**Supplementary Table 14. Performance on KIBA in random task split (Few-shot)**

| **Methods** | **MSE** | **CI** | **R2** | **Spearman** | **Pearson** |
| --- | --- | --- | --- | --- | --- |
| DeepDTA | 0.7534  (0.1138) | 0.5860  (0.0232) | -0.0290  (0.1088) | 0.2391  (0.0665) | 0.2606  (0.0639) |
| ColdDTA | 0.7023  (0.1385) | 0.5866  (0.0310) | 0.0363  (0.0652) | 0.2370  (0.0836) | 0.2379  (0.0890) |
| Co-VAE | 0.7100  (0.1048) | 0.6327  (0.0172) | 0.0844  (0.0491) | 0.3577  (0.0472) | 0.4127  (0.0412) |
| HiSIF | 0.7193  (0.1309) | 0.5914  (0.0124) | 0.0256  (0.0206) | 0.2517  (0.0330) | 0.2247  (0.0306) |
| MetaDTA | 0.6383  (0.1185) | 0.6150  (0.0508) | 0.1210  (0.0544) | 0.3097  (0.0457) | 0.3563  (0.0497) |
| CML | 0.7713  (0.1363) | 0.6272  (0.0253) | -0.0463  (0.0864) | 0.3457  (0.0669) | 0.2636  (0.0686) |
| PSICHIC | 0.8755  (0.1587) | 0.6169  (0.0476) | 0.0732  (0.1053) | 0.3276  (0.0474) | 0.2899  (0.0431) |
| ZeroBind | 0.7054  (0.1402) | 0.6127  (0.0572) | 0.0068  (0.0628) | 0.2849  (0.0555) | 0.3118  (0.0892) |
| AdaMBind(ours) | **0.5461**  **(0.0900)** | **0.6891**  **(0.0255)** | **0.2540**  **(0.0757)** | **0.5051**  **(0.0631)** | **0.5219**  **(0.0666)** |

The best results are highlighted in bold, while the second-best results are underlined. Five independent replications of each method were performed (n = 5). Data are expressed as means (std).

**Supplementary Table 15. Performance on BindingDB in random task split (Few-shot)**

| **Methods** | **MSE** | **CI** | **R2** | **Spearman** | **Pearson** |
| --- | --- | --- | --- | --- | --- |
| DeepDTA | 1.517  (0.1462) | 0.6812  (0.0476) | 0.1683  (0.0796) | 0.4334  (0.1211) | 0.4224  (0.1005) |
| ColdDTA | 1.3474  (0.2696) | 0.7042  (0.0342) | 0.2390  (0.0652) | 0.4858  (0.0800) | 0.5022  (0.0566) |
| Co-VAE | 1.3554  (0.2501) | 0.6818  (0.0213) | 0.1958  (0.0282) | 0.4237  (0.0254) | 0.4701  (0.0332) |
| HiSIF | 1.4322  (0.1762) | 0.7252  (0.0346) | 0.2215  (0.0735) | 0.4839  (0.0814) | 0.5103  (0.0819) |
| MetaDTA | 1.4342  (0.1907) | **0.7534**  **(0.0110)** | 0.2693  (0.0338) | 0.5011  (0.0436) | 0.5282  (0.0405) |
| CML | 1.3982  (0.1728) | 0.6965  (0.0356) | 0.2029  (0.0941) | 0.4637  (0.0862) | 0.5050  (0.0780) |
| PSICHIC | 1.7273  (0.2463) | 0.6880  (0.0369) | 0.1696  (0.0264) | 0.4612  (0.0420) | 0.4378  (0.0452) |
| ZeroBind | 1.3503  (0.1573) | 0.7007  (0.0486) | 0.2177  (0.0410) | 0.4623  (0.0718) | 0.5009  (0.0645) |
| AdaMBind(ours) | **1.2116**  **(0.1255)** | 0.7260  (0.0292) | **0.3094**  **(0.0629)** | **0.5323**  **(0.0758)** | **0.5634**  **(0.0590)** |

The best results are highlighted in bold, while the second-best results are underlined. Five independent replications of each method were performed (n = 5). Data are expressed as means (std).

**Supplementary Table 16. Performance on Davis in novel task split (Majority)**

| **Methods** | **MSE** | **CI** | **R2** | **Spearman** | **Pearson** |
| --- | --- | --- | --- | --- | --- |
| DeepDTA | 0.6230  (0.2530) | 0.7853  (0.0335) | 0.3346  (0.0997) | 0.5560  (0.0425) | 0.6097  (0.0691) |
| ColdDTA | 0.5731  (0.2184) | 0.7940  (0.0180) | 0.3809  (0.0914) | 0.5722  (0.0261) | 0.6316  (0.0613) |
| Co-VAE | **0.4960**  **(0.1809)** | **0.8187**  **(0.0312)** | 0.4168  (0.0561) | 0.5704  (0.0206) | 0.6517  (0.0402) |
| HiSIF | 0.6407  (0.2614) | 0.7887  (0.0325) | 0.3215  (0.0723) | 0.5673  (0.0654) | 0.5966  (0.0533) |
| MetaDTA | 0.6714  (0.1728) | 0.8163  (0.0228) | 0.4156  (0.0681) | **0.6606**  **(0.0263)** | 0.6454  (0.0584) |
| CML | 0.6301  (0.2050) | 0.7680  (0.0141) | 0.3117  (0.0798) | 0.5288  (0.0525) | 0.5592  (0.0723) |
| ZeroBind | 0.6506  (0.1524) | 0.7582  (0.0202) | 0.2882  (0.0361) | 0.5248  (0.0378) | 0.5713  (0.0405) |
| PSICHIC | 0.8463  (0.1891) | 0.7739  (0.0432) | 0.2915  (0.0543) | 0.5668  (0.0515) | 0.6051  (0.0581) |
| AdaMBind(ours) | 0.5080  (0.1823) | 0.8066  (0.0271) | **0.4460**  **(0.0808)** | 0.5978  (0.0633) | **0.6702**  **(0.0623)** |

The best results are highlighted in bold, while the second-best results are underlined. Five independent replications of each method were performed (n = 5). Data are expressed as means (std).

**Supplementary Table 17. Performance on KIBA in novel task split (Majority)**

| **Methods** | **MSE** | **CI** | **R2** | **Spearman** | **Pearson** |
| --- | --- | --- | --- | --- | --- |
| DeepDTA | 0.5160  (0.1091) | 0.6512  (0.0346) | 0.1409  (0.1374) | 0.3985  (0.0845) | 0.4857  (0.0661) |
| ColdDTA | 0.4851  (0.0713) | 0.7140  (0.0207) | 0.2821  (0.0101) | 0.5224  (0.0140) | 0.5352  (0.0191) |
| Co-VAE | 0.5159  (0.0836) | 0.6595  (0.0241) | 0.1071  (0.1229) | 0.4194  (0.0559) | 0.4781  (0.0394) |
| HiSIF | 0.4754  (0.0911) | 0.6632  (0.0524) | 0.2105  (0.0906) | 0.4260  (0.1290) | 0.4669  (0.0995) |
| MetaDTA | 0.5800  (0.0201) | 0.6184  (0.0375) | 0.0092  (0.1476) | 0.3170  (0.0981) | 0.3568  (0.1190) |
| CML | 0.5332  (0.0773) | 0.6605  (0.0121) | 0.1120  (0.0458) | 0.4289  (0.0274) | 0.3698  (0.0476) |
| ZeroBind | 0.5398  (0.0630) | 0.6461  (0.0330) | 0.1567  (0.0349) | 0.3928  (0.0699) | 0.4647  (0.0723) |
| PSICHIC | 0.4680  (0.0537) | 0.6411  (0.0344) | 0.1113  (0.0425) | 0.3702  (0.0609) | 0.3602  (0.0581) |
| AdaMBind(ours) | **0.3626**  **(0.0291)** | **0.7495**  **(0.0300)** | **0.3738**  **(0.0865)** | **0.6279**  **(0.0673)** | **0.6239**  **(0.0570)** |

The best results are highlighted in bold, while the second-best results are underlined. Five independent replications of each method were performed (n = 5). Data are expressed as means (std).

**Supplementary Table 18. Performance on BindingDB in novel task split (Majority)**

| **Methods** | **MSE** | **CI** | **R2** | **Spearman** | **Pearson** |
| --- | --- | --- | --- | --- | --- |
| DeepDTA | 1.2675  (0.2743) | 0.7081  (0.0348) | 0.1749  (0.0734) | 0.4754  (0.0682) | 0.4639  (0.0820) |
| ColdDTA | 0.8902  (0.1479) | 0.7412  (0.0740) | 0.3837  (0.1207) | 0.5810  (0.0541) | 0.6175  (0.0566) |
| Co-VAE | 1.1189  (0.3321) | 0.7447  (0.0370) | 0.2571  (0.1614) | 0.5481  (0.0886) | 0.5991  (0.0709) |
| HiSIF | 1.0064  (0.1391) | 0.7385  (0.0510) | 0.2846  (0.0745) | 0.5873  (0.0622) | 0.6102  (0.0541) |
| MetaDTA | 0.9987  (0.3440) | **0.7710**  **(0.0386)** | 0.3496  (0.1823) | **0.6086**  **(0.0924)** | 0.6313  (0.0876) |
| CML | 1.0545  (0.068) | 0.7323  (0.0260) | 0.2987  (0.0964) | 0.5287  (0.0993) | 0.5567  (0.0805) |
| ZeroBind | 1.2703  (0.0543) | 0.7274  (0.0282) | 0.3349  (0.0387) | 0.5391  (0.0689) | 0.6001  (0.00758) |
| PSICHIC | 1.634  (0.0721) | 0.7146  (0.0254) | 0.2185  (0.0233) | 0.5328  (0.0600) | 0.5282  (0.0640) |
| AdaMBind(ours) | **0.8758**  **(0.0374)** | 0.7689  (0.0348) | **0.4114**  **(0.1199)** | 0.6064  (0.1205) | **0.6407**  **(0.0869)** |

The best results are highlighted in bold, while the second-best results are underlined. Five independent replications of each method were performed (n = 5). Data are expressed as means (std).

| **Methods** | **MSE** | **CI** | **R2** | **Spearman** | **Pearson** |
| --- | --- | --- | --- | --- | --- |
| DeepDTA | 0.6283  (0.1924) | 0.7459  (0.0205) | 0.2202  (0.0641) | 0.4687  (0.0304) | 0.4946  (0.0517) |
| ColdDTA | 0.6464  (0.2420) | 0.7373  (0.0410) | 0.2145  (0.0898) | 0.4503  (0.0477) | 0.4795  (0.0928) |
| Co-VAE | **0.6069**  **(0.0784)** | 0.7114  (0.0055) | 0.1536  (0.0335) | 0.4043  (0.0061) | 0.4358  (0.0227) |
| HiSIF | 0.6682  (0.2303) | 0.7412  (0.0178) | 0.1811  (0.0727) | 0.4618  (0.0257) | 0.4784  (0.0630) |
| MetaDTA | 0.7664  (0.1250) | **0.7784**  **(0.0192)** | 0.2347  (0.0512) | 0.4749  (0.0341) | 0.5224  (0.0339) |
| CML | 0.7499  (0.1971) | 0.6556  (0.0190) | 0.0627  (0.0580) | 0.3028  (0.0582) | 0.2957  (0.0655) |
| ZeroBind | 0.7723  (0.1458) | 0.6285  (0.0251) | 0.0935  (0.0434) | 0.3260  (0.0535) | 0.3135  (0.0454) |
| PSICHIC | 0.7207  (0.0710) | 0.7023  (0.0467) | 0.2295  (0.0875) | 0.4086  (0.0611) | 0.4535  (0.0652) |
| AdaMBind(ours) | 0.6128  (0.2205) | 0.7591  (0.0186) | **0.2526**  **(0.0839)** | **0.4926**  **(0.0224)** | **0.5350**  **(0.0821)** |

**Supplementary Table 19. Performance on Davis in novel task split (Few-shot)**

The best results are highlighted in bold, while the second-best results are underlined. Five independent replications of each method were performed (n = 5). Data are expressed as means (std).

**Supplementary Table 20. Performance on KIBA in novel task split (Few-shot)**

| **Methods** | **MSE** | **CI** | **R2** | **Spearman** | **Pearson** |
| --- | --- | --- | --- | --- | --- |
| DeepDTA | 0.6932  (0.0784) | 0.6017  (0.0200) | -0.0118  (0.0973) | 0.2775  (0.0571) | 0.2937  (0.0576) |
| ColdDTA | 0.6532  (0.1324) | 0.6241  (0.0253) | 0.1055  (0.0222) | 0.3340  (0.0316) | 0.3709  (0.0074) |
| Co-VAE | 0.8888  (0.1119) | 0.5380  (0.0304) | -0.2686  (0.1119) | 0.1065  (0.0747) | 0.1121  (0.0861) |
| HiSIF | 0.9372  (0.1452) | 0.5681  (0.0534) | 0.0514  (0.1161) | 0.1937  (0.0952) | 0.2229  (0.0841) |
| MetaDTA | 0.6887  (0.1761) | 0.6451  (0.0303) | -0.0342  (0.4096) | 0.3864  (0.0816) | 0.4341  (0.0854) |
| CML | 0.7863  (0.1335) | 0.5992  (0.0418) | -0.1383  (0.0940) | 0.2675  (0.1068) | 0.2296  (0.0798) |
| ZeroBind | 0.7316  (0.1217) | 0.5549  (0.0330) | 0.0011  (0.1039) | 0.2324  (0.0643) | 0.2681  (0.0612) |
| PSICHIC | 0.9733  (0.1584) | 0.5375  (0.0462) | -0.1230  (0.1729) | 0.1078  (0.0547) | 0.1158  (0.0798) |
| AdaMBind(ours) | **0.4910**  **(0.1403)** | **0.6808**  **(0.0442)** | **0.2983**  **(0.1101)** | **0.4717**  **(0.0971)** | **0.5543**  **(0.0900)** |

The best results are highlighted in bold, while the second-best results are underlined. Five independent replications of each method were performed (n = 5). Data are expressed as means (std).

**Supplementary Table 21. Performance on BindingDB in novel task split (Few-shot)**

| **Methods** | **MSE** | **CI** | **R2** | **Spearman** | **Pearson** |
| --- | --- | --- | --- | --- | --- |
| DeepDTA | 1.6661  (0.3281) | 0.6627  (0.0167) | 0.0511  (0.1197) | 0.3875  (0.0282) | 0.3730  (0.0591) |
| ColdDTA | 1.6958  (0.1829) | 0.6813  (0.0353) | 0.1737  (0.0830) | 0.4387  (0.0833) | 0.4235  (0.0827) |
| Co-VAE | 1.3085  (0.1902) | 0.6779  (0.0348) | 0.0681  (0.0553) | 0.3996  (0.0841) | 0.3936  (0.0692) |
| HiSIF | 1.5272  (0.1517) | 0.6715  (0.0227) | 0.1233  (0.0449) | 0.4489  (0.0535) | 0.4756  (0.0519) |
| MetaDTA | 1.3613  (0.3461) | **0.7240**  **(0.0436)** | 0.2184  (0.1404) | 0.4508  (0.1788) | 0.4718  (0.1715) |
| CML | 1.4191  (0.2578) | 0.6926  (0.0232) | 0.1898  (0.0859) | 0.4529  (0.0451) | 0.5012  (0.0631) |
| ZeroBind | **1.2610**  **(0.2843)** | 0.6817  (0.0382) | 0.1443  (0.0534) | 0.4519  (0.0501) | 0.4656  (0.0269) |
| PSICHIC | 1.3137  (0.3719) | 0.6741  (0.0413) | 0.1807  (0.1144) | 0.4250  (0.0746) | 0.4689  (0.0739) |
| AdaMBind(ours) | 1.2968  (0.2460) | 0.6980  (0.0185) | **0.2536**  **(0.0370)** | **0.4703**  **(0.0477)** | **0.5171**  **(0.0557)** |

The best results are highlighted in bold, while the second-best results are underlined. Five independent replications of each method were performed (n = 5). Data are expressed as means (std).

**
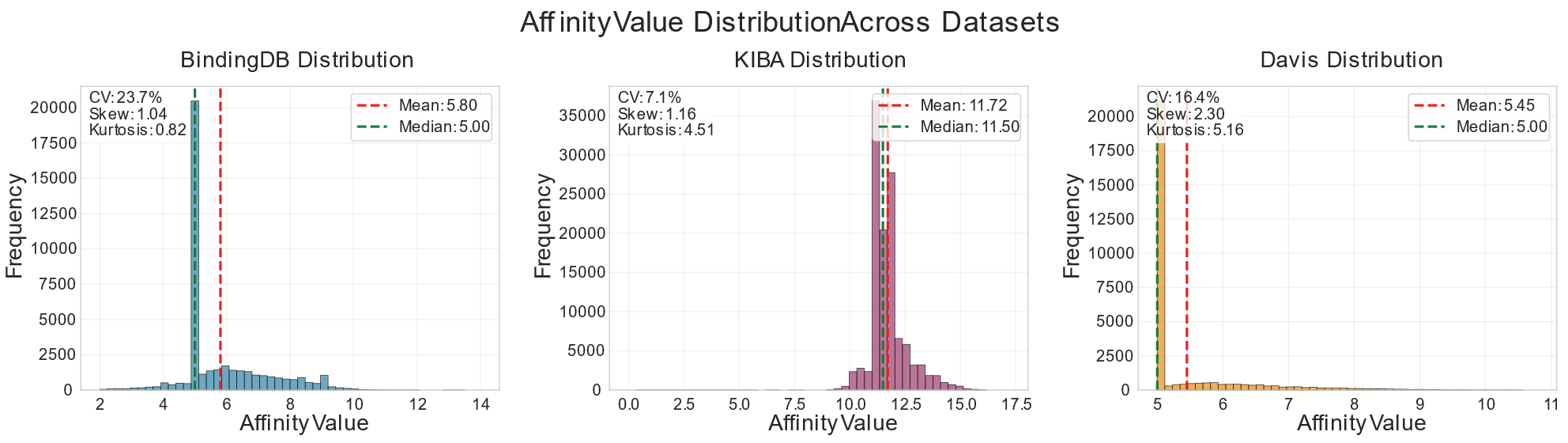
**

**Supplementary Fig.1** Affinity distribution across Davis, KIBA, and BindingDB datasets. The KIBA dataset (CV=7.14%) exhibits a highly concentrated distribution, with an interquartile range (IQR) of only 0.72, indicating that half of the data points fall within the narrow interval [11.20, 11.92]. Similarly, the Davis dataset (CV=16.41%) shows an even smaller IQR of 0.52, with data heavily concentrated between [5.00, 5.52]. In contrast, the BindingDB dataset (CV=23.65%) demonstrates a relatively moderate concentration. All three datasets are right-skewed (skewness > 0), with skewness values of 2.30 for Davis, 4.51 for KIBA, and 5.16 for BindingDB. Additionally, both the KIBA (kurtosis = 4.51) and Davis (kurtosis = 5.16) datasets display leptokurtic distributions (kurtosis > 3).

.
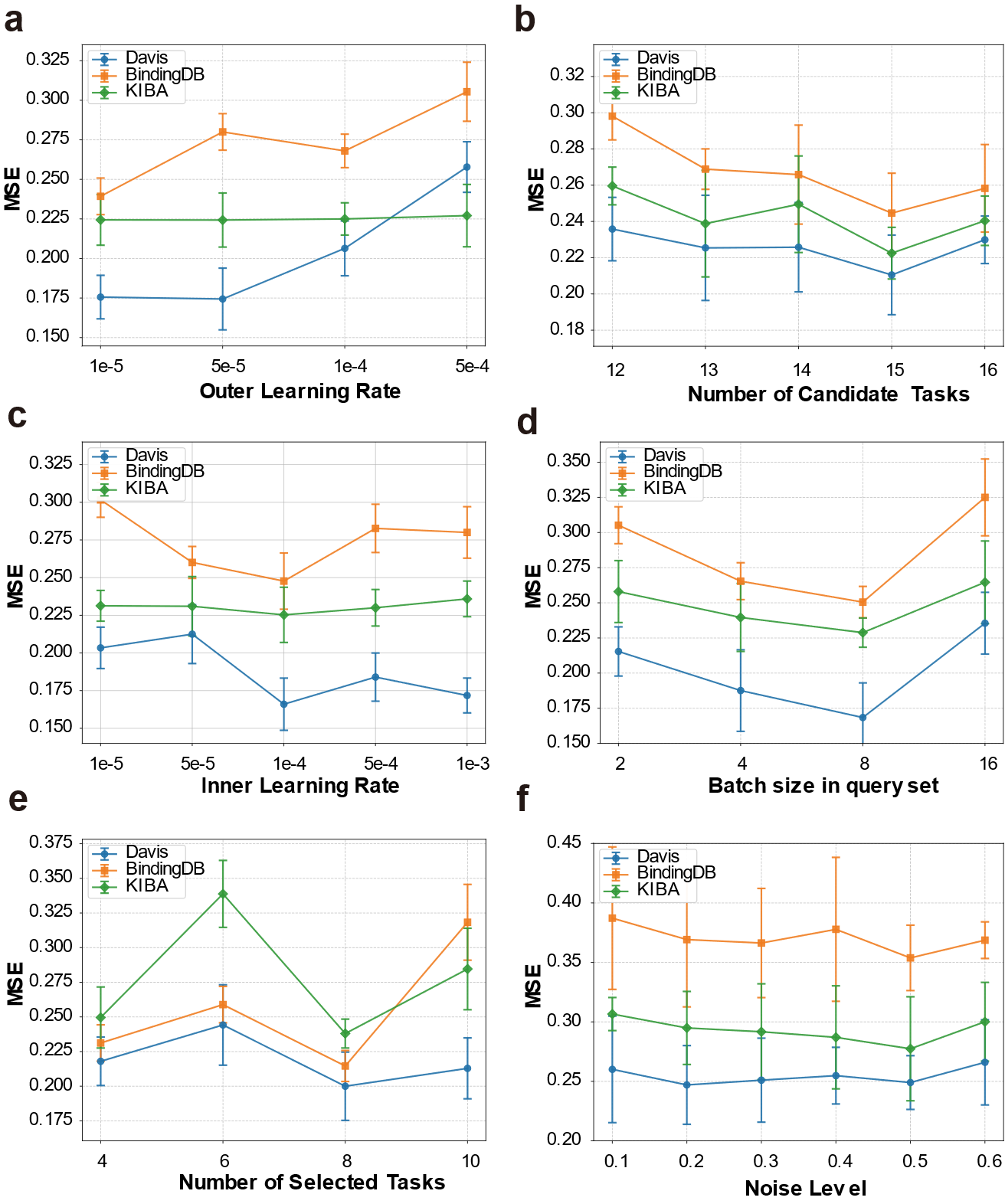


**Supplementary Fig.2** Impact of hyperparameters. Impact of outer learning rate (a), number of candidate tasks (b), inner learning rate (c), batch size in query set (d), number of selected tasks (e) and noise (f) on MSE across Davis, KIBA, BindingDB datasets. For the key hyperparameters of each major component (e.g., noise, outer learning rate, inner learning rate, batch size in the support set, number of candidate tasks, and number of selected tasks), we adopted a grid search strategy to systematically evaluate different combinations. First, we jointly assessed the influence of the inner learning rate and the support set batch size on model performance. Next, we explored the combined effect of the outer learning rate and the number of candidate tasks. Finally, we investigated the impact of noise and the number of selected tasks on model performance, as measured by MSE.


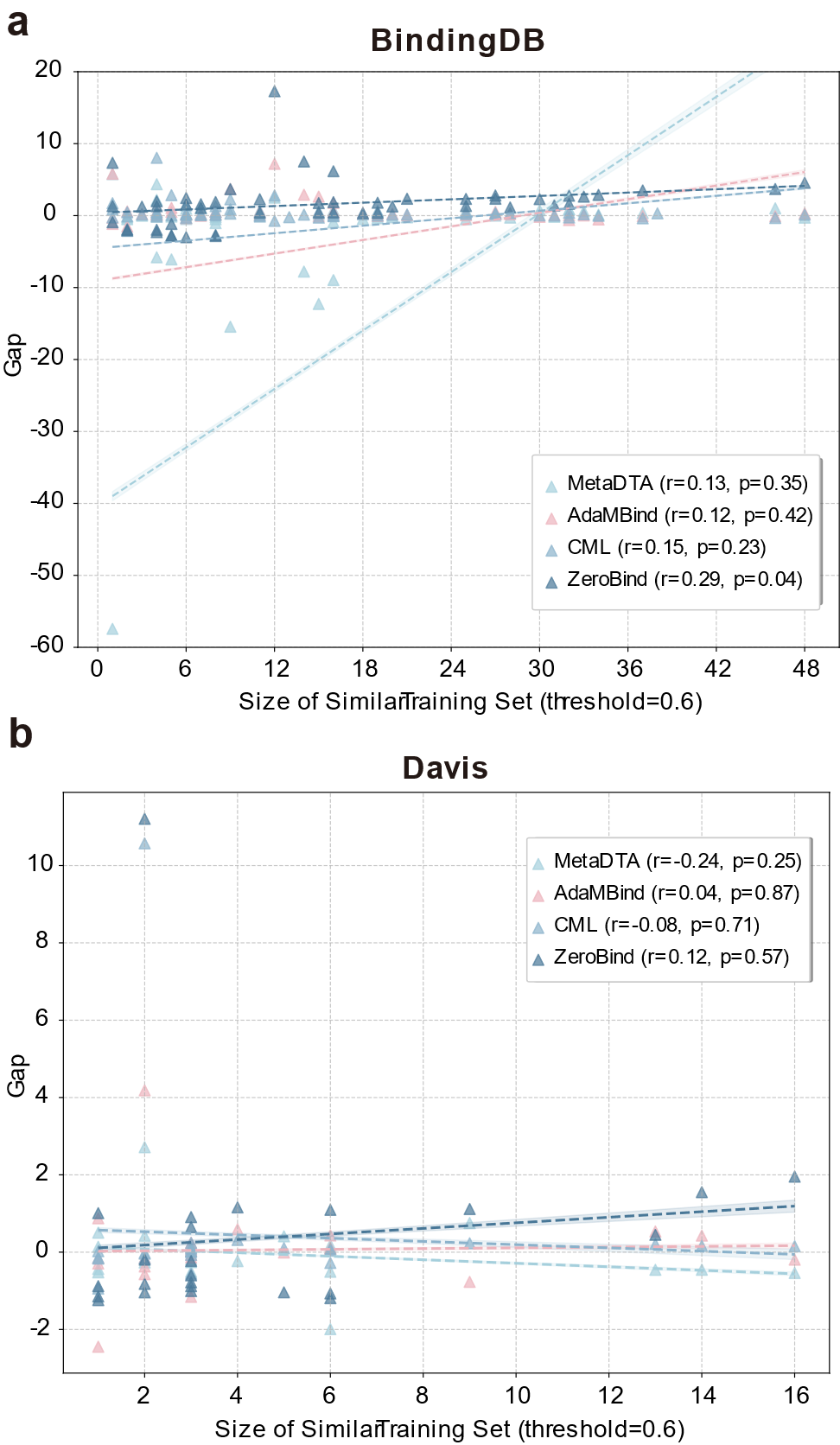


**Supplementary Fig.3** AdaMBind’s Generalization Performance in Relation to Task Similarity. This figure illustrates the relationship between the generalization gap ($Gap$) and the size of the similar training set ($N_{sim}$) on BindingDB (a) and Davis (b). Each point represents a test task. The x‑axis indicates the number of training tasks whose similarity to the current test task exceeds a threshold of 0.6. The y‑axis represents the generalization gap. For each model, the dashed line in the figure represents the least‑squares linear fit to the data points (two-sided Student test), and the shaded band denotes the 95% confidence interval of the regression. The slope of the fitted line quantifies how the generalization gap changes with the number of similar training tasks$N_{sim}$: a positive slope indicates that the gap widens (performance becomes more dependent on similarity) as more similar tasks become available, whereas a negative slope implies the opposite trend. A smaller absolute value of the generalization gap indicates that the model's performance is minimally influenced by task similarity.

| **Supplementary Algorithm 1.** AdaMBind training process |
| --- |
| **Require:** learning rates$\alpha$,$\beta$,training set $\{T_{i}\}_{i=1}^{N_{tr}}$, validation set $\{T_{i}\}_{i=1}^{N_{val}}$, candidate task size$B$, selected task set size$N_{slct}$ |
| 1: Initialize the meta learner $\theta_{0}^{(0)}$ and the adaptor $\varphi^{(0)}$ |
| 2: **for** k-th meta update **do**: |
| 3: Randomly sample B tasks from training set$\{T_{i}\}_{i=1}^{N_{tr}}$to form candidate task  set$\{T_{i}\}_{i=1}^{B}$ |
| 4: **for** each task$T_{i}$in candidate task set **do**: |
| 5: Initialize task-specific base leaner with $\theta_{0}^{(k)}$ |
| 6: update base learner via several steps, compute $Loss(Q_{i};\theta_{i}^{(k)})$and $\left\langle\nabla_{\theta_{0}^{(k)}}Loss(Q_{i}\right.;\theta_{0}^{(k)}),\left.\nabla_{\theta_{0}^{(k)}}Loss(S_{i};\theta_{0}^{(k)})\right\rangle$ |
| 7: generate sampling probability $\omega_{i}^{(k)}$by Eqn.(1) |
| 8: **end for** |
| 9: sample$N_{slct}$via sampling probability to form selected task set $\{T_{i}\}_{i=1}^{N_{slct}}$ |
| 10: compute approximate$\theta_{0}^{*}(\varphi)$by one -step gradient strategy as Eqn.(3) |
| 11: Randomly sample B tasks from validation set$\{T_{i}\}_{i=1}^{N_{val}}$, update adaptor $\varphi^{(k+1)}$ by Eqn.(2) |
| 12: generate sampling probabilityfor candidate task set by Eqn.(1) via using  updated$\varphi^{(k+1)}$ |
| 13: Resample another$N_{slct}$tasks to form selected task set $\{T_{i}^{'}\}_{i=1}^{N_{slct}}$ |
| 14: update meta learner $\theta_{0}^{(k+1)}$ by Eqn.(4) |
| 15: **end for** |

During the k-th meta update, for candidate task$T_{i}\in\{T_{i}\}_{i=1}^{B}$and meta learner $\theta_{0}^{(k)}$, we can define an adaptor $g_{\varphi}(\cdot)$ that generates task sampling weights $\omega_{i}$ with parameter $\varphi$:

$\omega_{i}^{(k)}=g\left(Loss(Q_{i};\theta_{i}^{(k)}),\left\langle\nabla_{\theta_{0}^{(k)}}Loss(Q_{i}\right.;\theta_{0}^{(k)}),\left.\nabla_{\theta_{0}^{(k)}}Loss(S_{i};\theta_{0}^{(k)})\right\rangle;\varphi^{(k)}\right)$ (1)

The update and optimization of the adaptor$\varphi$ can be described by the following formula:

$\varphi^{(k+1)}=\varphi^{(k)}-\beta\cdot\nabla_{\varphi}E_{T_{val}\sim p(T_{val})}[Loss(T_{val};\tilde{\theta}_{0}^{(k+1)}(\varphi^{(k)})]$ (2) $\theta_{0}^{*}(\varphi)\approx\tilde{\theta}_{0}^{(k+1)}(\varphi^{(k)})=\theta_{0}^{(k)}-\beta\cdot E_{T_{slct}\sim p(T_{slct})}[\nabla_{\theta}Loss(T_{slct};\theta_{0}^{(k)},\varphi^{(k)})]$ (3)

The update and optimization of the meta learner$\varphi^{(k+1)}$ can be described by the following formula:

$\theta_{0}^{(k+1)}(\varphi^{(k)})=\theta_{0}^{(k)}-\beta\cdot E_{T_{slct}^{'}\sim p(T_{slct}^{'})}[\nabla_{\theta}Loss(T_{slct}^{'};\theta_{0}^{(k)},\varphi^{(k+1)})]$ (4)
