# Supplementary material for: A meta learning and task adaptive approach for drug target affinity prediction
Source: Nat Commun. 2026 Mar 10;17:3734. doi: 10.1038/s41467-026-70554-5 (PMC13102954; doi:10.1038/s41467-026-70554-5)
Supplement: Supplementary file 3 — Reporting Summary [file 41467_2026_70554_MOESM3_ESM.pdf]

nature portfolio

Corresponding author(s): Xiaochen Bo

Last updated by author(s): Feb 13, 2026

Reporting Summary

Nature Portfolio wishes to improve the reproducibility of the work that we publish. This form provides structure for consistency and transparency in reporting. For further information on Nature Portfolio policies, see our Editorial Policies and the Editorial Policy Checklist.

Statistics

For all statistical analyses, confirm that the following items are present in the figure legend, table legend, main text, or Methods section.

n/a	Confirmed	
☐	☒	The exact sample size (*n*) for each experimental group/condition, given as a discrete number and unit of measurement
☐	☒	A statement on whether measurements were taken from distinct samples or whether the same sample was measured repeatedly
☐	☒	The statistical test(s) used AND whether they are one- or two-sided<br>*Only common tests should be described solely by name; describe more complex techniques in the Methods section.*
☒	☐	A description of all covariates tested
☐	☒	A description of any assumptions or corrections, such as tests of normality and adjustment for multiple comparisons
☐	☒	A full description of the statistical parameters including central tendency (e.g. means) or other basic estimates (e.g. regression coefficient) AND variation (e.g. standard deviation) or associated estimates of uncertainty (e.g. confidence intervals)
☐	☒	For null hypothesis testing, the test statistic (e.g. *F*, *t*, *r*) with confidence intervals, effect sizes, degrees of freedom and *P* value noted<br>*Give P values as exact values whenever suitable.*
☒	☐	For Bayesian analysis, information on the choice of priors and Markov chain Monte Carlo settings
☐	☒	For hierarchical and complex designs, identification of the appropriate level for tests and full reporting of outcomes
☐	☒	Estimates of effect sizes (e.g. Cohen's *d*, Pearson's *r*), indicating how they were calculated

Our web collection on statistics for biologists contains articles on many of the points above.

Software and code

Policy information about availability of computer code

Data collection	The source data and codes of AdaMBind is provided in https://github.com/Moohyun-w/AdaMBind, which has also been deposited in the Zenodo via 10.5281/zenodo.18595084
Data analysis	The source code and data of this study are available at https://github.com/Moohyun-w/AdaMBind. The specific version of the code associated with this publication is archived in Zenodo and is accessible via 10.5281/zenodo.1859508465. Data are analyzed using numpy v2.2.6 (https://numpy.org/), pandas v2.3.2 (https://pandas.pydata.org/), Seaborn V0.13.2 (https://seaborn.pydata.org/). Structures are visualized by Pymol v3.1.6 (https://www.pymol.org/) and LigPlot64 v2.1 (https://www.ebi.ac.uk/). Molecular docking simulations are performed using AutoDock4 v4.2.6 (https://autodock.scripps.edu/)

For manuscripts utilizing custom algorithms or software that are central to the research but not yet described in published literature, software must be made available to editors and reviewers. We strongly encourage code deposition in a community repository (e.g. GitHub). See the Nature Portfolio guidelines for submitting code & software for further information.

Data

Policy information about availability of data

All manuscripts must include a data availability statement. This statement should provide the following information, where applicable:

- Accession codes, unique identifiers, or web links for publicly available datasets
- A description of any restrictions on data availability
- For clinical datasets or third party data, please ensure that the statement adheres to our policy

The source data of three datasets used to train and evaluate the model is provided in https://github.com/Moohyun-w/AdaMBind/tree/main/data. The source data of LIT-PCBA dataset is provided in https://drugdesign.unistra.fr/LIT-PCBA/. The support set data used to construct the FLT3 inhibitor prediction task are available at https://doi.org/10.5281/zenodo.1363539363. Source data are provided with this paper through https://doi.org/10.6084/m9.figshare.30963823.

Research involving human participants, their data, or biological material

Policy information about studies with human participants or human data. See also policy information about sex, gender (identity/presentation), and sexual orientation and race, ethnicity and racism.

Reporting on sex and gender	N/A
Reporting on race, ethnicity, or other socially relevant groupings	N/A
Population characteristics	N/A
Recruitment	N/A
Ethics oversight	N/A

Note that full information on the approval of the study protocol must also be provided in the manuscript.

Field-specific reporting

Please select the one below that is the best fit for your research. If you are not sure, read the appropriate sections before making your selection.

[x] Life sciences   [ ] Behavioural & social sciences   [ ] Ecological, evolutionary & environmental sciences

For a reference copy of the document with all sections, see nature.com/documents/nr-reporting-summary-flat.pdf

Life sciences study design

All studies must disclose on these points even when the disclosure is negative.

Sample size	we trained our model using three widely recognized benchmark datasets: BindingDB, KIBA, and Davis. The Davis dataset includes kinase dissociation constant binding affinities between 68 kinase inhibitors and 442 target proteins, total 30056 DTAs. The KIBA dataset contains binding affinity data for 2,111 drugs and 229 target proteins, total 118254 DTAs. The BindingDB dataset comprises binding affinities for 9,864 drugs and 1,088 protein targets, total 42203 DTAs. Additionally, in experiments with limited data, we used the LIT-PCBA dataset proposed by Li et al., which includes 15 targets, 7844 active compounds, and 407,381 inactive compounds.
Data exclusions	In Davis,KIBA and BindingDB datasets, SMILES strings that the RDkKit python package could not recognize were removed.
Replication	To ensure statistical robustness and reproducibility, all reported results were obtained from five independent experimental runs, each executed with a distinct random seed.
Randomization	Random task split: where all tasks are randomly partitioned into meta-training, meta-validation, and meta-testing task sets at a ratio of 8:1:1. Novel task split: where tasks are partitioned based on protein sequence similarity using CD-HIT at a 40% identity threshold, proteins with sequence identity ≥ 40% were grouped into the same cluster, while those with identity < 40% were placed into different clusters. The resulting clusters were then allocated to meta-training, meta-validation, and meta-testing sets in an 8:1:1 ratio. This ensures that proteins from different sets share low sequence similarity (< 40%) and belong to distinct clusters. Consequently, the meta-testing tasks contain targets that are structurally and sequentially dissimilar to those encountered during meta-training.
Blinding	The investigators were blinded to group allocation during experiments and outcome analysis.

Reporting for specific materials, systems and methods

We require information from authors about some types of materials, experimental systems and methods used in many studies. Here, indicate whether each material, system or method listed is relevant to your study. If you are not sure if a list item applies to your research, read the appropriate section before selecting a response.

Materials & experimental systems

n/a	Involved in the study
☒	☐ Antibodies
☒	☐ Eukaryotic cell lines
☒	☐ Palaeontology and archaeology
☒	☐ Animals and other organisms
☒	☐ Clinical data
☒	☐ Dual use research of concern
☒	☐ Plants

Methods

n/a	Involved in the study
☒	☐ ChIP-seq
☒	☐ Flow cytometry
☒	☐ MRI-based neuroimaging

Plants

Seed stocks	N/A
Novel plant genotypes	N/A
Authentication	N/A
